# Non operative management of liver and spleen traumatic injuries: a giant with clay feet

**DOI:** 10.1186/1749-7922-7-3

**Published:** 2012-01-23

**Authors:** Salomone Di Saverio, Ernest E Moore, Gregorio Tugnoli, Noel Naidoo, Luca Ansaloni, Stefano Bonilauri, Michele Cucchi, Fausto Catena

**Affiliations:** 1 Maggiore Hospital - Bologna Local Health District Trauma Surgery Unit (Head Dr. G. Tugnoli) Department of Emergency, Department of Surgery L.go Nigrisoli, ZIP 40123, Bologna, Italy; 2 Trauma Services, Rocky Mountain Regional Trauma Center at Denver Health Medical Center Department of Surgery, Denver Health Medical, Center, University of Colorado Health Sciences Center Department of Surgery, University of Colorado Health Sciences Center, Denver, USA; 3 General Surgery I, Ospedali Riuniti, Bergamo, Italy; 4 Emergency Surgery Unit Department of General and Transplant surgery (Prof. A. D. Pinna) S. Orsola Malpighi University Hospital Via Massarenti, 40138, Bologna, Italy; 5 Charlotte Maxeke Johannesburg Academic Hospital, Department of Surgery, University of Witwatersand Medical School, Johannesburg, South Africa; 6 Reggio Emilia Hospital, Reggio Emilia, Italy

## 

After years of initial aggressive surgical intervention and a subsequent shift to damage control surgery (DCS), non operative management (NOM) has been shown to be safe and effective.

In fact trauma surgeons realized that in liver trauma, it was safer to pack livers [1] than do finger fracture [2] or resection, and this represented a tangential issue to nonoperative approach.

Damage control was not the paradigm shift for spleen and liver, but rather to address coagulopathy that was more commonly associated with penetrating major abdominal vascular injuries [3].

The shift to nonoperative care was largely motivated by intraoperative observations that many minor liver [4] and splenic injuries [5] were found no longer bleeding.

Then CT arrived in the early 1980s and confirmed that many moderate liver and spleen injuries did not require OR intervention. Pediatric surgeons first lead the shift to nonoperative management for splenic trauma [6,7].

In the 90's it became the gold standard for liver injuries in hemodynamically stable patients, regardless of injury grade and degree of hemoperitoneum [8], allowing better outcomes with fewer complications and lesser transfusions [9]. Nevertheless concerns have been raised regarding continuous monitoring required [10], safety in higher grades of injury [11] and general applicability of NOM to all haemodynamically stable patients [12]. Similarly, in the same period and following promising results obtained with splenic salvage [13] with several surgical techniques [14] such as splenorraphy, high intensity ultrasound, haemostatic wraps and staplers [15], NOM became the treatment of choice for blunt splenic injuries [5]. However it was immediately clear that NOM failure in adults was significantly higher than that observed in children (17% vs 2%). The incidence of immune system sequelae, coupled with Overwhelming Post Surgical Infection (OPSI) and their real clinical impact, is difficult to establish in the overall population including children [16].

Although recent reports [17] showed that despite a similar incidence and severity of solid organ injuries, Trauma centers with higher risk-adjusted mortality rates are more likely to undertake operative interventions for solid organ injuries. Data from The American College of Surgeons' National Trauma Data Bank including 87,237 solid abdominal organ injuries showed that, despite a strongly significant increase in percentage of NOM for hepatic and splenic trauma, mortality has remained unchanged [18].

More recently several authors have highlighted an excessive use of NOM, which for some high grade liver injuries is pushed far beyond the reasonable limits, carrying increased morbidity at short and long term, such as bilomas, biliary fistulae, early or late haemorrhage, false aneurysm, arteriovenous fistulae, haemobilia, liver abscess, and liver necrosis [19]. Incidence of complications attributed to NOM increases in concert with the grade of injury. In a series of 337 patients with liver injury grades III-V treated non-operatively, those with grade III had a complication rate of 1%, grade IV 21%, and grade V 63% [20]. Patients with grades IV and V injuries are more likely to require operation, and to have complications of non-operative treatment. Therefore, although it is not essential to perform liver resection at the first laparotomy, if bleeding has been effectively controlled [21], increasing evidence suggests that liver resection should be considered as a surgical option in patients with complex liver injury, as an initial or delayed strategy, which can be accomplished with low mortality and liver related morbidity in experienced hands [22].

Liver resection in hepatic trauma should be considered when (1) massive bleeding related to a hepatic venous injury, (2) massive destruction and devitalized hepatic tissue is present, often partially resected by the injury itself, or (3) a major bile leak coming from a proximal, main intrahepatic biliary duct are found.

NOM of liver injuries grade > = 3, especially when treated with combined AngioEmbolization (AE), is not without risks (mainly biliary leaks, liver necrosis and severe sepsis) and may lead to significant morbidity and possible mortality in up to 11% of cases due to liver-related complications [23].

Although AE has been defined the logical augmentation of damage control techniques for controlling hemorrhage, the overall liver-related complication rate can be as high as 60.6% with 42.2% incidence of Major Hepatic Necrosis [24]. Early liver lobectomy in such cases required lesser number of procedures and achieved lower complication rate and lower mortality compared to less aggressive approaches such as serial operative debridements and/or percutaneous drainage [25].

Further concerns for both liver and spleen NOM, arise when associated hepatic and splenic injuries coexist and/or potentially missed injuries can be suspected. Patients with associated liver and spleen injuries are twice as likely to fail non-operative therapy as those with only a single organ injured [26]. Missing associated intra-abdominal injury and delayed treatment, significantly affects the outcome. This occurs more often in conjunction with liver than with splenic injury, especially pancreas and bowel injury are significantly associated with liver injury in blunt trauma [27].

NOM is actually used blunt splenic as the initial standard of care for blunt splenic injuries, not only in children (rates above 90-95%) but also in adults (60-77% [28]). Even in Grade IV-V splenic injuries NOM attempt has been pushed up to in 40.5% but it ultimately failed in 55% of these high-grade injuries [29]. This is despite the fact that, already in the late 90's, it became clear that significant numbers of delayed splenic complications occurred with nonoperative management of splenic injuries which were potentially life-threatening [30].

A significantly higher failure rate (38%) has been observed in grade IV-V Blunt Splenic injury(BSI) patients and above all, mortality of patients for whom NOM failed was almost 7-fold higher than those with successful NOM in this series (4.7% vs 0.7%; *p *= .07) [31]. Furthermore, multivariate analysis identified 2 independent predictors of f-NOM: grade V BSI and the presence of a brain injury. Other authors identified age > 55 years, ISS > 25 and lower level trauma centers admission as predictors of splenic NOM failure [32]. That means NOM should be carefully initiated in severe grade of BSI and careful selection of candidates for NOM is advisable for a safe conservative management choice.

In the most recent years a liberal and more aggressive use of angiography has often been observed and is associated with higher rates of NOM (80%) and lower rates of failure (2-5%); nonetheless several concerns raise because it is labour intensive and there have been several reports reporting a surprisingly high rate of complications [33]. In WTA multi-institutional experience, among 140 patients underwent AE, 27 (20%) suffered major complications including 16 (11%) failure to control bleeding (requiring 9 splenectomies and 7 repeat AE), 4 (3%) missed injuries, 6 (4%) splenic abscesses, and 1 iatrogenic vascular injury [34]. Additionally, proximal splenic artery embolization (SAE), has been introduced in an attempt to increase overall success rates of NOM in high grade BSI, but the following has been observed: (1) high failure rates of proximal SAE in all patients with grade V injuries and the majority of grade IV injuries, (2) the immunologic consequences of proximal SAE are unclear, and whether its use provides true salvage of splenic function versus simple avoidance of operative splenectomy, (3) an increased incidence of Adult Respiratory Distress Syndrome (ARDS). This was 4-fold higher in those patients that underwent proximal SAE compared with those that underwent operative splenectomy (22% vs. 5%, *p *= 0.002). Higher rates of septic complications including splenic abscess, septicemia, and pneumonia have also been recorded, and lastly (4) a non significant trend to higher amount of PRBC (packed red blood cell) transfusions, higher mortality and longer Length Of Stay [35].

Splenic preservation can also have deleterious side effects in otherwise salvageable patients. A review of 78 patients who failed NOM revealed a mortality rate of 12.6%. The authors concluded that the majority of their deaths were a result of delayed treatment of intra-abdominal injuries, and suggested that 70% of deaths after failing NOM were potentially preventable [36]. When extrapolated to a large series like the EAST trial, this means that 33 unnecessary deaths occurred or 0.5% of all patients treated non-operatively. Compared to a death rate from OPSI of 1/10,000 adult splenectomised patients, the odds are 20 times greater that a patient would die from failure of NOMSI than from OPSI [37].

Thus we surgeons must keep in our minds that post-splenectomy sepsis is rare and can be minimized with polyvalent vaccines of encapsulated bacteria, whilst operative mortality of splenectomy in the otherwise normal patient is < 1% [38].

Whereas Non Operative Management of Liver Injury (NOMLI) has not been shown to increase mortality rates for those that fail, the same cannot be said for the NOMSI and the balance between concerns with bleeding and infection has in the most recent years shifted illogically to favour infection. As Richardson highlighted, it should be made clear that these delayed bleeding and late failures of NOM are not harmful. "Anecdotally, I have been impressed in private discussions about deaths or "near misses" from bleeding occurring in NOM failures. These are rarely reported in the literature. Additionally, many reports list multiple organ failure as a leading cause of death. Does unrecognized shock play a role in these deaths?" [39].

In conclusion, at the beginning of the 21^st ^century, when NOM for liver and spleen injuries is often advocated beyond the limits of a reasonable safety and the need for surgery is considered as a defeat or "failure". We should not forget in making the best treatment choice, to keep in mind not only the predictors of NOM failure, such as the injury grade, the presence of associated intra-abdominal injuries and the risk of missing injuries with the subsequent sequelae, of a failed NOM and of delayed surgical treatment, but we must also consider the potential drawbacks of angioembolization, the environmental setting and factors, i.e. the level of the hospital (trauma center), availability of Angio Suite and ICU for continuous monitoring, the initiation of NOM during night shift, the need of an eventual time consuming spine surgery in a prone position for a concomitant vertebral fracture, and last but not least, the time needed for complete and safe resumption of normal life (work and physical activity).

## References

[B1] Feliciano DV Mattox KL Jordan GL Intra-abdominal packing for control of hepatic hemorrhage: a reappraisal J Trauma 1981 21 285 290 10.1097/00005373-198104000-00005 7012380

[B2] Pachter HL Spencer FC Hofstetter SR Coppa GF Experience with the finger fracture technique to achieve intra-hepatic hemostasis in 75 patients with severe injuries of the liver Ann Surg 1983 197 6 771 8 10.1097/00000658-198306000-00017 6344818 PMC1352914

[B3] Stone HH Strom PR Mullins RJ Management of the major coagulopathy with onset during laparotomy Ann Surg 1983 197 5 532 5 10.1097/00000658-198305000-00005 6847272 PMC1353025

[B4] Lucas CE Ledgerwood AM Changing times and the treatment of liver injury Am Surg 2000 66 4 337 41 10776869

[B5] Cogbill TH Moore EE Jurkovich GJ Nonoperative management of blunt splenic trauma: a multicenter experience J Trauma 1989 29 1312 1317 10.1097/00005373-198910000-00002 2681805

[B6] Pearl RH Wesson DE Spence LJ Filler RM Ein SH Shandling B Superina RA Splenic injury: a 5-year update with improved results and changing criteria for conservative management J Pediatr Surg 1989 24 1 121 4 disc 124-5 10.1016/S0022-3468(89)80315-X 2723984

[B7] Rothenberg S Moore EE Marx JA Moore FA McCroskey BL Selective management of blunt abdominal trauma in children--the triage role of peritoneal lavage J Trauma 1987 27 10 1101 6 10.1097/00005373-198710000-00001 3669104

[B8] Pachter HL Knudson MM Esrig B Status of nonoperative management of blunt hepatic injuries in 1995: a multicenter experience with 404 patients J Trauma 1996 40 31 38 10.1097/00005373-199601000-00007 8576995

[B9] Croce MA Fabian TC Menke PG Waddle-Smith L Minard G Kudsk KA Patton JH Jr Schurr MJ Pritchard FE Nonoperative management of blunt hepatic trauma is the treatment of choice for hemodynamically stable patients. Results of a prospective trial Ann Surg 1995 221 6 744 53 discussion 753-5 10.1097/00000658-199506000-00013 7794078 PMC1234706

[B10] Knudson MM Lim RC Jr Oakes DD Jeffrey RB Jr Nonoperative management of blunt liver injuries in adults: the need for continued surveillance J Trauma 1990 30 1494 1500 10.1097/00005373-199012000-00009 2258960

[B11] Sherman HF Savage BA Jones L Nonoperative management of blunt hepatic injuries: safe at any grade? J Trauma 1994 37 616 621 10.1097/00005373-199410000-00015 7932893

[B12] Meredith JW Young JS Bowling J Roboussin D Nonoperative management of blunt hepatic trauma: the exception or the rule? J Trauma 1994 36 529 534 discussion 534-535 10.1097/00005373-199404000-00012 8158715

[B13] Pickhardt B Moore EE Moore FA McCroskey BL Moore GE Operative splenic salvage in adults: a decade perspective J Trauma 1989 29 1386 1391 10.1097/00005373-198910000-00017 2810416

[B14] Millikan JS Moore EE Moore GE Stevens RE Alternatives to splenectomy in adults after trauma. Repair, partial resection, and reimplantation of splenic tissue Am J Surg 1982 144 6 711 6 10.1016/0002-9610(82)90556-6 7149130

[B15] Lucas CE Splenic trauma. Choice of management Ann Surg 1991 213 2 98 112 10.1097/00000658-199102000-00002 1992948 PMC1358380

[B16] Passlick B Izbicki J Waydhas C Nast-Kolb D Schweiberer L Ziegler-Heitbrock H Posttraumatic splenectomy does not influence human peripheral blood mononuclear cell subsets J Clin Lab Immunol 1991 34 4 157 61 1668282

[B17] Shafi S Parks J Ahn C Gentilello LM Nathens AB More operations, more deaths? Relationship between operative intervention rates and risk-adjusted mortality at trauma centers J Trauma 2010 69 1 70 7 10.1097/TA.0b013e3181e28168 20622580

[B18] Hurtuk M Reed RL Esposito TJ Davis KA Luchette FA Trauma surgeons practice what they preach: The NTDB story on solid organ injury management J Trauma 2006 61 2 243 54 discussion 254-5 10.1097/01.ta.0000231353.06095.8d 16917435

[B19] Trunkey DD Hepatic trauma: contemporary management Surg Clin North Am 2004 84 2 437 50 10.1016/S0039-6109(03)00228-7 15062654

[B20] Kozar RA Moore JB Niles SE Complications of non-operative management of high-grade blunt hepatic injuries J Trauma 2005 59 1066 1071 16385280 10.1097/01.ta.0000188937.75879.ab

[B21] Piper GL Peitzman AB Current management of hepatic trauma Surg Clin North Am 2010 90 4 775 85 10.1016/j.suc.2010.04.009 20637947

[B22] Polanco P Leon S Pineda J Puyana JC Ochoa JB Alarcon L Harbrecht BG Geller D Peitzman AB Hepatic resection in the management of complex injury to the liver J Trauma 2008 65 6 1264 9 discussion 1269-70 10.1097/TA.0b013e3181904749 19077611

[B23] Goldman R Zilkoski M Mullins R Mayberry J Deveney C Trunkey D Delayed celiotomy for the treatment of bile leak, compartment syndrome, and other hazards of nonoperative management of blunt liver injury Am J Surg 2003 185 5 492 7 10.1016/S0002-9610(03)00046-1 12727573

[B24] Dabbs DN Stein DM Scalea TM Major hepatic necrosis: a common complication after angioembolization for treatment of high-grade liver injuries J Trauma 2009 66 3 621 7 discussion 627-9 10.1097/TA.0b013e31819919f2 19276729

[B25] Dabbs DN Stein DM Philosophe B Scalea TM Treatment of major hepatic necrosis: lobectomy versus serial debridement J Trauma 2010 69 3 562 7 10.1097/TA.0b013e3181ebf591 20838127

[B26] Malhotra AK Latifi R Fabian TC Multiplicity of solid organ injury: influence on management and outcomes after blunt abdominal trauma J Trauma 2003 54 925 9 10.1097/01.TA.0000066182.67385.86 12777905

[B27] Miller PR Croce MA Bee TK Malhotra AK Fabian TC Associated injuries in blunt solid organ trauma: implications for missed injury in nonoperative management J Trauma 2002 53 2 238 42 discussion 242-4 10.1097/00005373-200208000-00008 12169928

[B28] Tinkoff G Esposito TJ Reed J Kilgo P Fildes J Pasquale M Meredith JW American Association for the Surgery of Trauma Organ Injury Scale I: spleen, liver, and kidney, validation based on the National Trauma Data Bank J Am Coll Surg 2008 207 5 646 55 10.1016/j.jamcollsurg.2008.06.342 18954775

[B29] Watson GA Rosengart MR Zenati MS Nonoperative management of severe blunt splenic injury: are we getting better? J Trauma 2006 61 1113 1118 discussion 1118-1119 10.1097/01.ta.0000241363.97619.d6 17099516

[B30] Cocanour CS Moore FA Ware DN Marvin RG Clark JM Duke JH Delayed complications of nonoperative management of blunt adult splenic trauma Arch Surg 1998 133 6 619 24 discussion 624-5 10.1001/archsurg.133.6.619 9637460

[B31] Velmahos GC Management of the most severely injured spleen: a multicenter study of the Research Consortium of New England Centers for Trauma (ReCONECT) Arch Surg 2010 145 5 456 60 10.1001/archsurg.2010.58 20479344

[B32] McIntyre LK Schiff M Jurkovich GJ Failure of nonoperative management of splenic injuries: causes and consequences Arch Surg 2005 140 6 563 8 discussion 568-9 10.1001/archsurg.140.6.563 15967903

[B33] Peitzman AB Richardson JD Surgical treatment of injuries to the solid abdominal organs: a 50-year perspective from the Journal of Trauma J Trauma 2010 69 5 1011 21 10.1097/TA.0b013e3181f9c216 21068605

[B34] Moore FA Davis JW Moore EE Jr Cocanour CS West MA McIntyre RC Jr Western Trauma Association critical decisions in trauma: management of adults splenic trauma J Trauma 2008 65 1007 1011 10.1097/TA.0b013e31818a93bf 19001966

[B35] Duchesne JC Simmons JD Schmieg RE Jr McSwain NE Jr Bellows CF Proximal splenic angioembolization does not improve outcomes in treating blunt splenic injuries compared with splenectomy: a cohort analysis J Trauma 2008 65 6 1346 51 discussion 1351-3 10.1097/TA.0b013e31818c29ea 19077625

[B36] Peitzman AB Harbrecht BG Rivera L Heil B Failure of observation of blunt splenic injury in adults: variability in practice and adverse consequences J Am Coll Surg 2005 201 179 187 10.1016/j.jamcollsurg.2005.03.037 16038813

[B37] Franklin GA Casós SR Current advances in the surgical approach to abdominal trauma Injury 2006 37 12 1143 56 Epub 2006 Nov 7 10.1016/j.injury.2006.07.018 17092502

[B38] Root HD Splenic injury: angiogram vs. operation J Trauma 2007 62 6 Suppl S27 17556952 10.1097/TA.0b013e318065403c

[B39] Richardson JD Changes in the management of injuries to the liver and spleen J Am Coll Surg 2005 200 5 648 69 Review 10.1016/j.jamcollsurg.2004.11.005 15848355

